# A Jackstone Calculus Residing in a Urinary Bladder Diverticulum

**DOI:** 10.7759/cureus.30140

**Published:** 2022-10-10

**Authors:** Kristophe M Anderson, Joseph C Herring

**Affiliations:** 1 Internal Medicine, Harlem Hospital Center, New York, USA; 2 Radiology, Abdominal Imaging, University of Washington, Seattle, USA

**Keywords:** vesical calculi, ct, jackstone, bladder diverticulum, jackstone calculus

## Abstract

Jackstone calculi are uncommon urinary stones, characterized by a central dense core with a spiculated contour, which resemble the toy from the children’s game Jacks. They are usually described in the veterinary literature. When present, they are typically found in the vesical bladder. There is limited literature about this unusual type of calculus. We describe a rare case of a Jackstone calculus, found inside a bladder diverticulum, detected on CT abdomen in a non-verbal patient with non-specific abdominal pain, chronic urinary retention, and microscopic hematuria.

## Introduction

A bladder diverticulum can be defined as a herniation of the bladder mucosa and urothelium when a defect is found between the detrusor muscle fibers, resulting in a thin-walled sac connected to the bladder lumen [1]. A Jackstone calculus is a rare subtype of urinary calculus with a spiculated appearance that bears a close resemblance to the objects used in the children’s game ‘Jacks’. This type of calculus usually consists of calcium oxalate dihydrate, unlike other types of renal calculi that typically consist of calcium oxalate monohydrate. It is essential to identify the characteristic shape of the Jackstones, as they are more easily fragmented by lithotripsy than calcium monohydrate stones [2].

## Case presentation

An 80-year-old man presented from a skilled nursing facility with fever, hypoxia, and a decline in mental status. Past medical history is significant for a right occipital hemorrhagic cerebrovascular accident with respiratory failure resulting in a tracheostomy and percutaneous endoscopic gastrostomy (PEG) tube placement, atrial fibrillation, and chronic urinary retention with indwelling foley catheter secondary to prostatomegaly. One month prior to his presentation, he was admitted for gross hematuria and subsequently treated for UTI. The etiology of gross hematuria was to be worked up on a non-emergent outpatient basis following discharge.

On presentation, the patient was noted to be hypoxic, tachycardic, and tachypneic. On physical exam, the patient was alert but non-verbal and unable to obey commands. Coarse breath sounds were auscultated bilaterally, and facial grimacing was noted with deep palpation of the lower abdomen. The Foley catheter was changed and observed to be draining cloudy, amber urine. Initial laboratory workup was significant for a positive coronavirus disease 2019 (COVID-19) polymerase chain reaction (PCR) and acute kidney injury (AKI). The urinalysis was positive for leukocyte esterase and many WBCs and RBCs. Non-contrast CT of the brain revealed no acute changes.

The patient was subsequently admitted to the medical service for the management of acute hypoxic respiratory failure and metabolic encephalopathy secondary to COVID-19 pneumonia, as well as sepsis secondary to a urinary source likely in the setting of obstructive uropathy.

**Figure 1:**
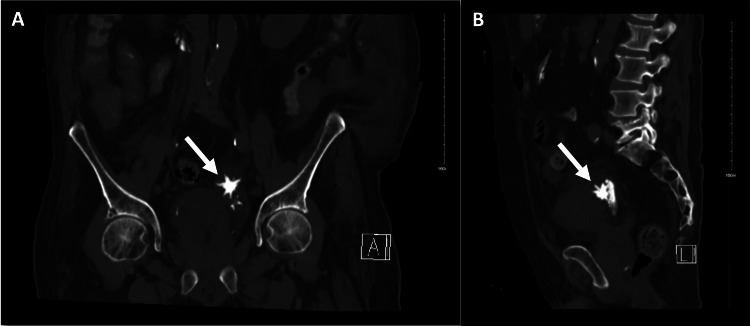
(A) coronal and (b) sagittal views of non-contrast CT abdomen showing spiculated Jackstone calculus (arrow) inside a bladder diverticulum

Due to the findings on the physical exam of the abdomen, a non-contrast CT of the abdomen was obtained, which revealed a moderately distended urinary bladder (despite a Foley catheter in place). Additionally, a large left vesical bladder wall diverticulum was discovered containing a 3.8 x 3.2 cm spiculated calculus with a mean attenuation of 900 Hounsfield Units (Figure 1). Adjacent fat stranding and diffuse bladder wall thickening were also present.

The patient was stabilized in the ICU and stepped down to the medical floor. Urology was consulted and recommended non-emergent, elective stone removal pending a family discussion for determining the goals of care. After clinical improvement, the patient was discharged to a skilled nursing facility and referred to the urology clinic for follow-up; however, he did not attend the scheduled appointment.

## Discussion

Jackstone calculi are urinary calculi that consist of irregular, spiculated contours bearing a close resemblance to a sea urchin or the toy “jacks” used in the traditional children’s game. Although typically located in the vesical bladder, they may also be found in the upper urinary tract, such as the renal pelvis, but this is less common [3,4]. Most urinary calculi consist of calcium oxalate crystals. Calculi consisting of calcium oxalate monohydrate crystals are typically smooth and black in color. On the other hand, Jackstone calculi consist predominantly of calcium oxalate dihydrate crystals, which tend to be irregular and yellow in color [2]. It is hypothesized that the spiky projections of calcium dihydrate crystals repeatedly make contact with the bladder wall, sloughing off the soft precipitated apatite and adherent mucoproteins while concurrently allowing the deposition of more calcium oxalate. As a result, the calculus grows mainly at the distal tips, producing the irregular, spiculated Jackstone shape from a previously mamillated stone [5].

Imaging evaluation of urinary calculi is most accurately assessed by CT due to its high sensitivity and specificity. The Hounsfield unit allows for detecting the homogeneity or heterogeneity structure of calculi. Dual-energy CT is a computed tomography technique that has been implemented in clinical practice since only 2006 [6]. This technique uses two separate X-ray photon energy spectra, allowing for the interrogation of materials that have different attenuation properties at different energies. Conventional single-energy CT is unable to accurately determine the chemical composition of stones, whereas dual-energy CT has been shown to accurately differentiate uric acid stones from non-uric acid stones, with a diagnostic accuracy ranging from 90% to 100%. Characterizing the chemical composition of stones is essential because uric acid stones may be treated with alkalinization of the urine while non-uric acid (e.g. calcium oxalate) require other therapeutic techniques such as mechanical fragmentation or percutaneous lithotomy [6,7]. Jackstone calculi are composed of calcium oxalate dihydrate with a loose crystalline structure. This composition makes them easily fragmented during lithotripsy. Therefore, identifying a Jackstone calculus has clinical and therapeutic implications [2,3,8].

**Figure 2:**
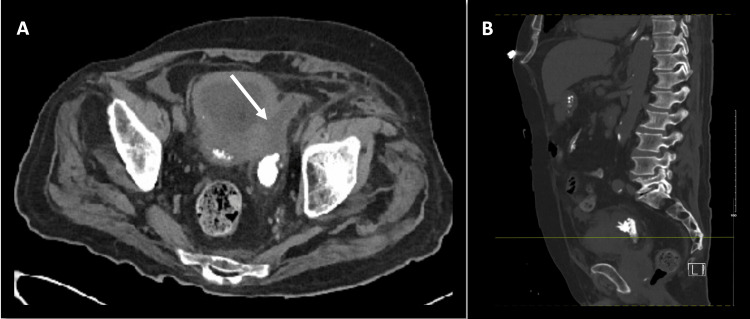
(A) axial and (B) sagittal views of non-contrast CT abdomen in the index patient, showing a thickened vesical bladder wall and large left bladder wall diverticulum (arrow) with intraluminal calculus in place posteriorly (the inferior aspect of the calculus shown in axial view)

In adults, a urinary bladder diverticulum is represented as a mucosal herniation, usually secondary to chronic outflow obstruction (Figure 2). Urinary stasis may precipitate stone formation, a complication that occurs in roughly 12% of diverticulae. Those that form in the diverticulum are usually a result of infection, particularly in urinary tract infections caused by urea-splitting organisms. Stones may also migrate into the diverticulum from the bladder and kidneys [9].

In this patient, the urine culture was positive for a non-urease-splitting organism, Escherichia (E.) coli. The urinary stasis brought about by the enlarged prostate and bladder diverticulum were both risk factors for the formation of the Jackstone calculus. Options for treatment include cystoscopy, percutaneous cystostomy, and open cystostomy with stone removal. Extracorporeal shockwave lithotripsy was not recommended because of the stone’s location in the pelvis. Diverticulectomy was not advised due to the invasive nature of the procedure for our patient who was non-ambulatory, with various co-morbidities.

Bladder calculus formation is a known complication of a urinary bladder diverticulum, however, to our knowledge, there have been no reported cases in the literature of Jackstone calculi within a diverticulum.

## Conclusions

Jackstone calculi consist of calcium oxalate dihydrate and have a unique shape, resembling a sea urchin or the toy ‘Jacks’. CT is highly sensitive and specific in identifying these calculi and, due to their composition, they are highly susceptible to fragmentation by lithotripsy. They are typically found in the vesical bladder, limiting the efficacy of lithotripsy, and less commonly in the upper urinary tract. Calculi may form in a bladder diverticulum as a complication of infection from a urease-splitting organism or by migration from the kidneys or vesical bladder, which is the likely mechanism in our patient.
